# Carrier-to-Noise-Threshold Filtering on Off-Shore Wind Lidar Measurements

**DOI:** 10.3390/s19030592

**Published:** 2019-01-30

**Authors:** Sven-Erik Gryning, Rogier Floors

**Affiliations:** DTU Wind Energy, Technical University of Denmark, Risø Campus, Roskilde, DK-4000, Denmark; rofl@dtu.dk

**Keywords:** Carrier-to-Noise-Ratio (CNR), heterodyne doppler wind lidar, off-shore, climatology wind speed, direction and power density

## Abstract

Wind lidar observations are characterized by a Carrier-to-Noise-Ratio that is often used to filter the observations. The choice of the Carrier-to-Noise-Ratio threshold value for the wind lidar observations is found to have an effect on the climatological wind speed distribution in such a way that when the Carrier-to-Noise-Ratio (CNR) threshold value is increased the wind speed distribution is shifted to higher values. Based on one year of observations carried out with a wind lidar from 126 m to 626 m height at the FINO3 (Forschungsplattform in Nord- und Ostsee Nr. 3) research platform in the North Sea, the effect that the choice of the Carrier-to-Noise threshold value has on the climatology of the wind speed and direction as well as the wind power density in relation to wind energy is illustrated and discussed. In the one-year data set considered here it is found that for thresholds larger than −29 dB, the mean wind speed and wind rose measured by the wind lidar become a function of the threshold value, and for values smaller than ~ −29 dB further decrease of the Carrier-to-Noise-Ratio threshold has a minor effect on the estimated mean wind speed and wind rose. The analysis of the data set from the North Sea shows that the limit for the Carrier-to-Noise-Ratio of the observations should be −29 dB or less to obtain a threshold independent estimate of the mean wind speed and wind rose. Alternatively, all valid observations should be used for the analysis. Although this study is specific for the conditions in the North Sea, we suggest that for a representative estimation of the wind resource with wind lidars, the effect of the CNR threshold filtering on the wind distribution should be studied when the recovery rate is less than 100%.

## 1. Introduction

In recent years off-shore wind energy has become a mature and commercially competitive technology for energy generation [1,2]. As the wind resource is highly variable both in time and space, several methods to estimate the wind energy resource have been developed. All the methods depend on trustworthy long time-series of profiles of the wind speed and direction. Traditionally such time series were obtained from well instrumented meteorological masts [3], but this has limited the observations and wind profile studies to about 100 m [4,5,6,7,8,9,10] in most cases, as only a few 200 to 300 m instrumented masts exists, e.g., the 280 m instrumented TV tower Hamburg [11,12], the 200 m meteorological mast at Cabauw in the Netherlands [13,14], and the 244 m tall meteorological mast at Østerild in Denmark, that became operational in 2015.

This limitation in height coverage from instrumented masts has resulted—albeit some mainly on trial basis—in the use of a number of remote sensing techniques in wind energy resource assessment studies, including satellites in the off-shore environment [15,16], dual-Doppler radar [17], and Doppler wind lidars [18,19]. To meet the requirements for resolution and accuracy that are needed for wind energy resource assessment, the Doppler wind lidar has become the remote sensing instrument of preference since its appearance as a commercially available unit, although still not fully recognized as a bankable alternative to the traditional wind speed measurements by cup-anemometers.

In this study, off-shore observations of the wind profile up to 626 m performed with a Doppler wind lidar are analyzed. A Doppler wind lidar can, under favorable conditions, reach higher up. Frehlich argued in [20] that if the so-called Carrier-to-Noise-Ratio (CNR) falls below a prescribed threshold value, the uncertainty in the wind speed is too large for the measurements to be useful; he recommended CNR >−22 dB. The uncertainty of the individual observations by the wind lidar depends on the CNR in such a way that high values of the CNR threshold value are associated with observations of predominantly high wind speeds with a low uncertainty and decreasing threshold values are associated with increasing uncertainty of the observed wind speed [21,22,23,24]. This observation will be further developed in this study.

A method to compensate for the instrument noise which reduces the signal-to-noise ratio threshold value that is used as a measure of the data quality is presented in [25]. Along this line [26] suggests a post-processing algorithm that provides a better estimate of the signal-to-noise-ratio of the observations, which results in a more accurate estimate of the uncertainty of the line-of-sight measurements. Presently the algorithm is implemented for a Halo Doppler Wind Lidar in Finland and is shown to improve the sensitivity of the observations with at least a factor of five.

Alternatives to the CNR threshold data filtering approach have been suggested by Suomi [9] and Beck and Kühn [27], based on dynamic data-filtering. Beck and Kühn demonstrated that the line-of-sight-velocity measurements performed by a long-range lidar over a horizontal distance of ~3 km over the sea can be dynamically filtered to maximize accuracy and data availability of mean radial velocities. The theory has not been implemented for the horizontal velocity in a vertical wind profile and will not be considered in this study.

## 2. Site and Instrumentation

**Figure 1:**
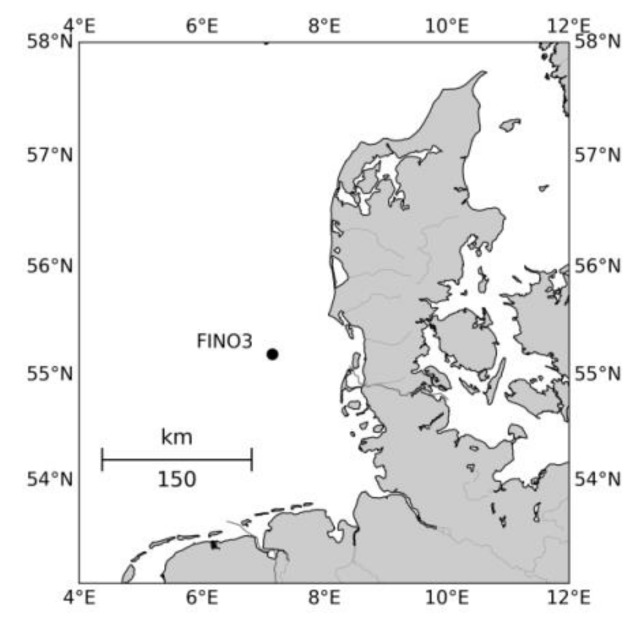
Map showing the position of the FINO3 (Forschungsplattform in Nord- und Ostsee Nr. 3) research platform where the measurements were carried out. Water is marked in white and land in grey.

The observations for this study have been collected off-shore in the North Sea at the German Research Platform FINO3 (Forschungsplattform in Nord- und Ostsee Nr. 3) located at (55.19501° N; 7.15836° E) 80 km west of the Danish island Rømø, Figure 1, at a water depth of 22 m. The approved offshore wind farms Sandbank 24, Nördlicher Grund, DanTysk, and OSB Butendiek lie in the near vicinity, but the construction of these was not yet initiated during the measuring campaign.

The measurements were carried out with a ~ 600 m range wind Doppler lidar in combination with a 100 m tall meteorological mast equipped with cup-anemometers at 50 m, 70 m, and 90 m height in combination with wind vanes at 28 and 100 m.

### 2.1. Wind Doppler Lidar

During the period 29 August 2013 to 4 October 2014 10 min averaged measurements were performed with a wind lidar (WLS70 made by Leosphere, a Vaisala Company, Saclay, France) installed at the platform 24.6 m above mean sea level. The wind lidar measured the radial wind velocity in four azimuth angles separated by 90° at a zenith angle of 15°. Starting from 100 m up to 2 km with a 50 m vertical resolution, horizontal wind velocities were determined. Details on the wind lidar and how it was operated during the campaign are provided by [22].

In a coherent-detection lidar, such as the one used in this study, the backscattered signal is mixed with a signal from a local oscillator beam in the lidar before being sensed by a photodetector. For each of the range gates, the resulting signal is processed in real time, and radial-velocity spectra are computed and averaged. The peak frequency in the velocity spectra is a measure of the Doppler shift, providing the radial Doppler velocities from which the wind speed is calculated.

The measurements from wind lidars rely on the presence of small airborne particles, such as aerosols, to act as tracers of the wind and provide a backscattering signal. The four most important atmospheric factors influencing the wind lidar performance are aerosol backscatter, relative humidity, precipitation, and atmospheric refractive turbulence [28]. 

Lidar-data quality is specified in terms of the strength of the heterodyne signal relative to the level of noise—the so-called Carrier-to-Noise-Ratio (CNR), details are given in [18,29,30]. The CNR is linearly proportional to the backscatter and inversely proportional to the square of the propagation distance. Therefore, data availability decreases with range when using a constant CNR value to filter the measurements. When the heterodyne signal is very weak, velocity estimates are dominated by noise and, thus, subject to estimation errors. If the CNR falls below a predetermined threshold, Frehlich [20] suggests that the uncertainty in the velocity is too large for the data to be useful. Therefore, it has become common practice to filter out measurements for this type of lidar when CNR <–22 dB, [28]. 

**Figure 2:**
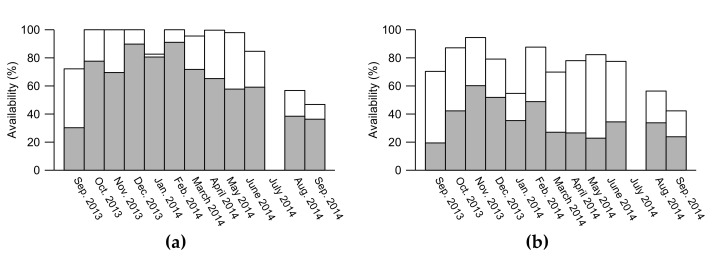
Monthly data coverage for Carrier-to-Noise-Ratio (CNR) >−35 dB (white plus grey) and CNR >−22 (grey only) at the 126 m level (**a**) and at all levels up to 626 m (**b**). 100 % availability corresponds to the number of 10-min periods in each month.

Some of the consequences of filtering the data with a prescribed CNR threshold value are discussed in [31] and are further elaborated on in this study. The wind lidar profiles used in this study were analyzed up to ~600 m above the wind lidar to ensure a sufficiently large dataset for the analysis. A full profile is identified when the CNR of concurrent measurements at all levels from 126 m to 626 m is above a threshold value. Some analysis is also performed for CNR threshold values at 126 m and 626 m only. The monthly data coverage is illustrated in Figure 2.

### 2.2. Cup Anemometer and Wind Vane Observations

The FINO3 platform is equipped with a 100 m lattice mast with booms for meteorological observations between 30 and 105 m above the mean sea level. In certain directions, however, the mast disturbs the flow, so three levels are equipped with cup anemometers on booms for each of the directions 105°, 225°, and 345° providing a less disturbed dataset of the 10 min averaged wind speeds. The procedure for selecting the corrected observations is provided in [32]. Wind directions are measured at 28 m and 100 m with wind vanes every 10 min.

## 3. Data Analysis 

In this section, the wind speed dependence on the CNR threshold value is discussed. All illustrations are shown for a height of 126 m above the mean sea level, corresponding to the lowest observation height of the wind lidar, and it is also the most relevant for today’s wind turbines. However, many are also illustrated for 626 m height. The analysis of the observations include all available data from the period September 2013 to September 2014, and, thus, represent a period of slightly more than a year.

### 3.1. Wind Speed Dependence on Carrier-to-Noise-Ratio

Contrary to measurements by a cup-anemometer, wind speed measurements performed by a wind lidar are assigned a quality indicator expressed as the Carrier-to-Noise-Ratio. Reference [31] pointed out a consequence of the choice of a CNR threshold value on the wind observations; increasing the threshold value increases the estimated mean wind speed. The effect was found over land, in coastal areas, and off-shore. The findings of Reference [31] will be extended to consider the effect that filtering of the data with a CNR threshold value has on the distribution of the wind speed and direction as well as the wind power density; the latter indicates how much power is available for conversion to energy by a wind turbine. 

**Figure 3:**
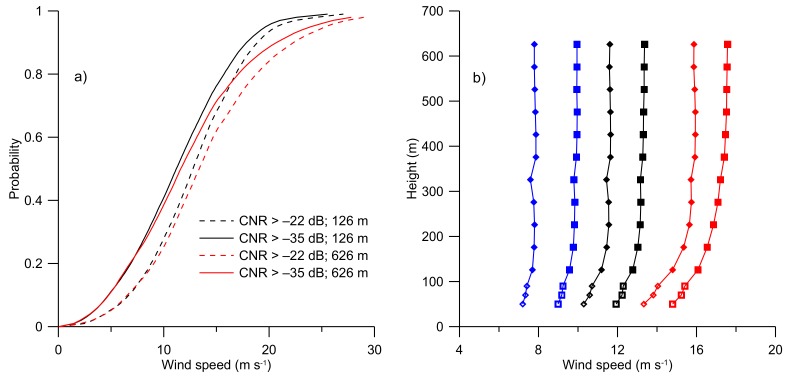
Panel (**a**) shows the cumulative distributions of wind speed observations. The full lines represent observations for a CNR threshold value of −35 dB, and dashed lines for −22 dB. Black lines represent measurements from 126 m height, and red lines from a height of 626 m. Panel (**b**) illustrates profiles of wind-speed percentiles for CNR >−35 dB (diamonds) and CNR >−22 dB (squares). The lines and symbols represent: blue 25%, black 50%, and red 75% percentiles. Solid symbols represent wind lidar measurements and open symbols mast observations.

To investigate how the filtering effect of the CNR threshold value acts on the wind speed data-series, Figure 3a shows the cumulative probability distribution of the wind speed observations at 126 and 626 m height. It can be observed that the wind-speed for the factory setting threshold value of −35 dB is always smaller as compared to −22 dB over the whole cumulative distribution at both heights. The effect is also illustrated for low, medium, and high wind speeds exemplified by the percentiles. This is illustrated in Figure 3b, showing the 25%, 50%, and 75% percentiles of the wind speed profiles for CNR threshold values of −22 dB and −35 dB, respectively. It can be observed that the profile of the wind speed is persistently larger for a CNR threshold value of −22 dB when compared to −35 dB. 

**Figure 4:**
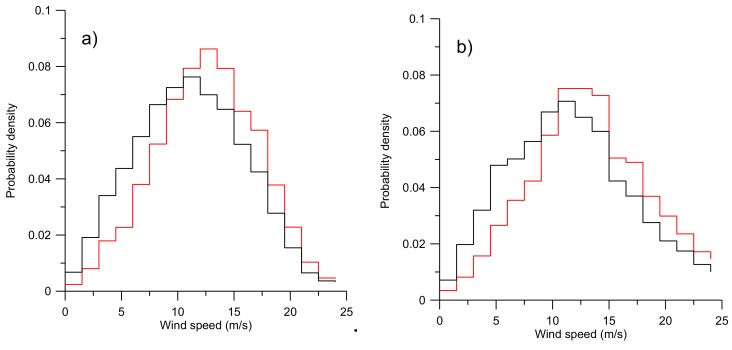
Wind speed distributions for (**a**) 126 m and (**b**) 626 m height. Red line represents the distribution for CNR >−22 dB and black for CNR >−35 dB, respectively. Note that the curves that are filtered with CNR >−22 dB (red) contain less data than the curves for CNR >−35 dB (black).

To further illustrate the effect on the choice of CNR threshold value, Figure 4 shows the wind speed distribution at 126 and 626 m height. It can be observed that the wind speed distribution is shifted towards higher values when applying the −22 dB threshold value as compared to a −35 dB threshold, thus, the filtering does not just remove low wind speed values but operates on all wind speeds in such a way that the wind speed distribution is shifted towards higher values. The effect can be observed at all heights covered by the campaign (50 to 626 m) and is here illustrated for heights of 126 and 626 m.

**Figure 5:**
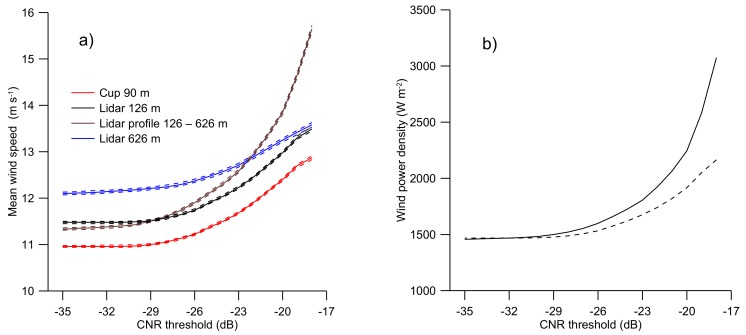
Panel (**a**) shows the mean wind speed plotted as a function of the CNR threshold. Full lines illustrate the relationship when filtering the measurements at 126 m (black), the concurrent cup anemometer measurements at 90 m (red), the measurements at 626 m (blue) with the CNR threshold value, and the full brown line when the CNR threshold is imposed at all levels between 126 m and 626 m. The uncertainty corresponding to the standard error of the mean wind speed is shown by dashed lines. The panel (**b**) illustrates the wind power density at 126 m height as a function of the CNR threshold value. The meaning of the line colors is similar in both panels. There are no measurements smaller than –35 dB which corresponds to the factory setting threshold value.

Figure 5 further illustrates the sensitivity of the mean wind speed and power density to the CNR threshold value. The effect is illustrated in Figure 5 a showing the mean wind speed (full lines) and the corresponding uncertainty (dashed lines) as a function of the CNR threshold value. The brown lines are when the CNR threshold is imposed on the full profile of lidar measurements up to 626 m, the other lines illustrate the relationship when the CNR threshold is not imposed on the full profile but only at the wind lidar measurements at 126 m (black), 626 m (blue), and the concurrent wind speed measurements from the cup anemometer at 90 m (red). The uncertainty is here taken as the standard error of the mean wind speed and is illustrated by the dashed lines above and below the mean wind speed. As pointed out by [31] for, e.g., lidar measurements at 126 m, being a typical height of a wind turbine, the use of a CNR threshold value of −22 dB in this off-shore case resulted in an overestimate of the mean wind speed of 12% for the full profile and 9% as compared to a CNR threshold value of –35 dB.

For the wind power density (Figure 5b) the effect is even more pronounced than for the wind speed, being 32% larger for a CNR threshold value of –22 dB compared to –35 dB when the threshold is imposed on the entire wind profile between 126 m and 626 m and 18% larger when the CNR threshold is applied to the observations at 126 m only. The wind power density was calculated by use of the Wind Atlas Analysis and Application Program (WAsP) [3] using an air density of 1.225 kg m^-3^. For wind energy assessment applications the Weibull distribution is commonly used to describe the variability of the wind speed. It is reported in [31] that filtering the wind speed observations with a CNR threshold also affects the shape parameter in the Weibull distribution.

**Figure 6:**
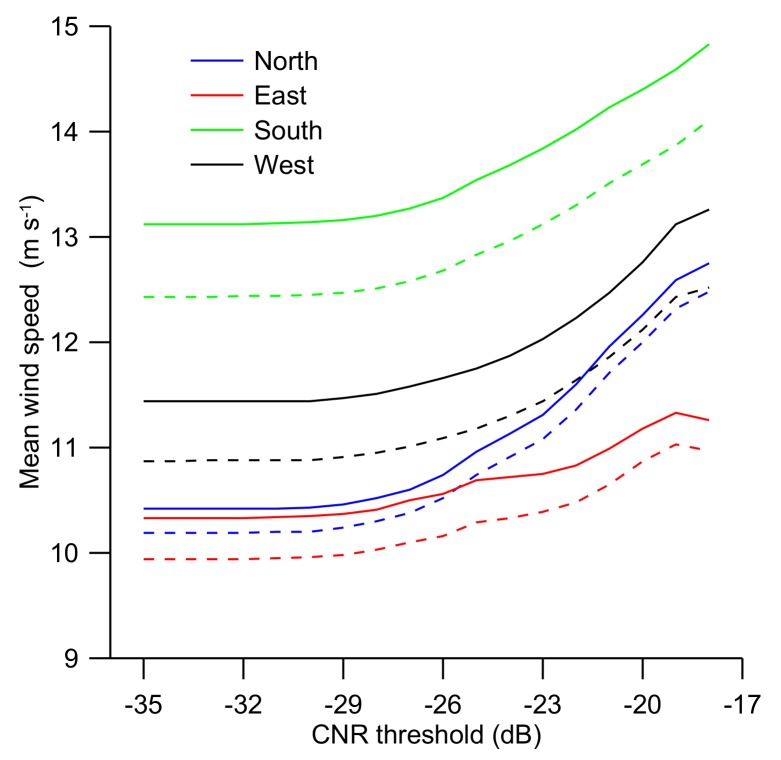
Mean wind speed as a function of the CNR threshold value for separate wind sectors as measured with the wind lidar at 126 m height (full lines) and the cup anemometer at 90 m height (dashed). Northern sector (325°–45°, blue lines), Easterly sector (45°–135°, red lines), Southerly sector (135°–225°, green lines) and Westerly sector (225°–345°, black lines).

To investigate if any wind direction dependency exists in the general relationship between the mean wind speed and the CNR threshold value, the mean wind speed was plotted for Northerly (325°–45°), Easterly (45°–135°), Southerly (135°–225°), and Westerly (225°–345°) wind directions (Figure 6). The relationship is illustrated for wind lidar measurements at 126 m (full lines) and the concurrent cup-anemometer observations at 90 m (dashed lines). A general tendency for the mean wind speed to decrease for decreasing CNR values can be observed in all the wind direction sectors, but for the eastern sector, the effect is not as pronounced as in the other sectors. High wind speeds in the western sectors and low wind speed in the eastern sector are typical for the meteorological conditions over the North Sea.

It can be observed from Figure 5 and Figure 6 that the mean wind speed decreases as the CNR threshold decreases from –17 dB to about –29 dB and then remains near constant as the CNR threshold is further decreased. This general behavior is observed in the wind lidar measurements at both 126 m, 626 m and for the concurrent cup anemometer observations at 90 m (Figure 5) as well as for all the wind direction sectors (Figure 6).

**Figure 7:**
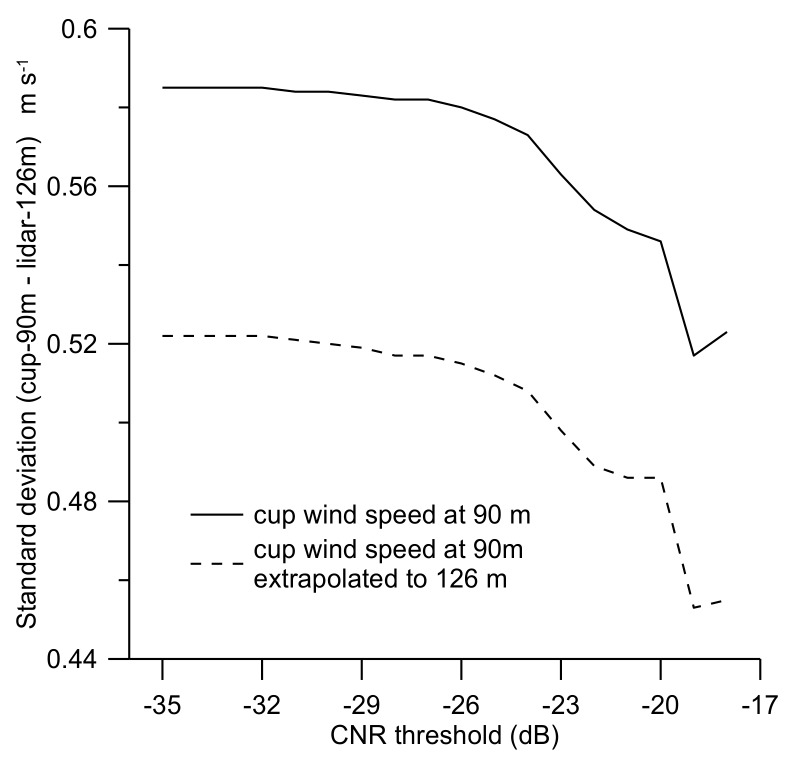
Standard deviation of the difference between the wind speed at 90 m ($u_{cup}\left(90\;m\right)$) and 126 m ($u_{lidar}\left(126\;m\right)$), $\sigma \left(u_{cup}\left(90\;m\right)-u_{lidar}\left(126\;m\right)\right)$, as a function of the CNR threshold. The full line illustrates the relationship without accounting for any wind shear between the measuring heights, the dashed line by use of the logarithmic wind profile to extrapolate the wind speed at 90 m to a height of 126 m.

Further investigation of the effect of the CNR threshold is performed by comparing the wind speed measured by the cup anemometer at 90 m with the wind lidar measurements at 126 m. Figure 7 shows the standard deviation of the difference between cup-anemometer (90 m) and the wind lidar (126 m) measurements of the wind speed. The full line is a direct comparison between the difference in wind speed between 90 m and 126 m, without accounting for the difference in wind speed between the two levels. For the dashed line the wind shear between 90 m and 126 m has been accounted for by extrapolation of the wind speed at 90 m to 126 m by use of the logarithmic wind profile, before forming the difference between the individual observations.

It can be observed that the standard deviation has a minimum at CNR ~ –19 dB, then it increases for decreasing CNR threshold values until a CNR of ~–29 dB and, thereafter, remains near constant as the CNR threshold is further decreased.

The comparison should ideally be performed with cup-anemometer and wind lidar measurements at the same height, but such measurements were not available here. By extrapolating the cup-anemometer measurements from 90 m to 126 m by assuming a logarithmic wind profile, the effect on the wind shear from the atmospheric stability is unaccounted for. Furthermore, the comparison is between point measurements for the cup anemometer with range averaged measurements for the wind lidar, which are not directly comparable.

### 3.2. Wind Direction Dependence on Carrier-to-Noise-Ratio

**Figure 8:**
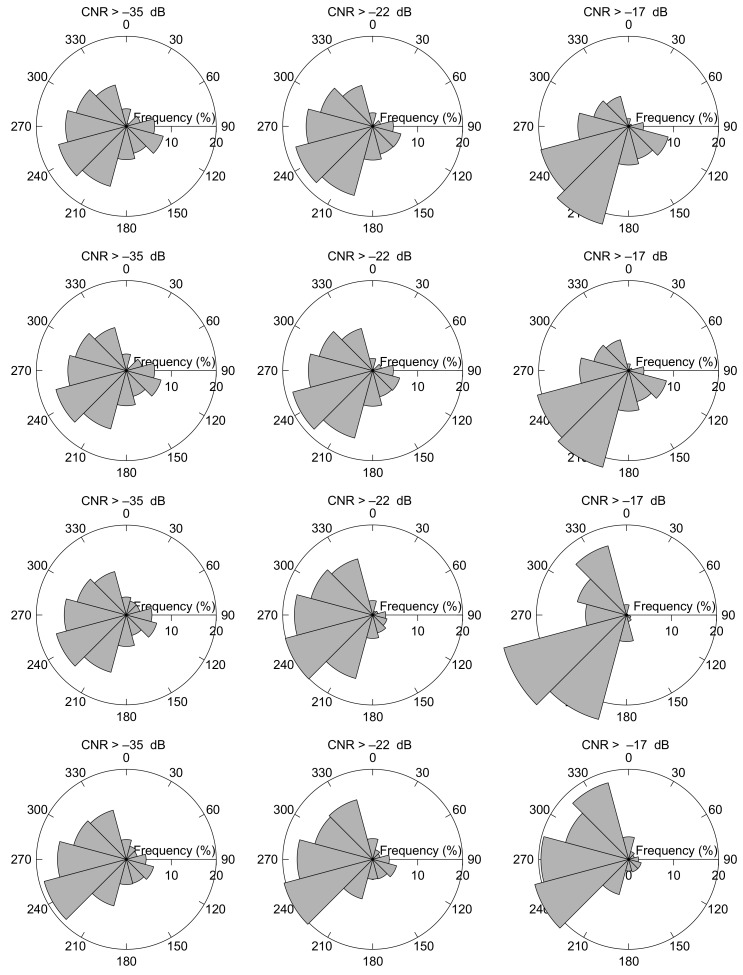
Wind roses at the FINO3 off-shore platform for CNR threshold values of –17 dB (right panels), –22 dB (middle panels) and –35 dB (left panels). The upper panels show the wind roses at 126 m when filtering the wind lidar measurements at 126 m height with the actual CNR threshold values, and the next highest panels are for concurrent wind vane observations at 100 m. The second lowest panels illustrate the wind roses at 126 m when the CNR threshold is applied at all levels between 126 m and 626 m, and the lowest panels the wind rose at 626 m when the CNR threshold is applied at 626 m only.

The wind direction climatology is also affected by the CNR threshold filtering. In Figure 8 wind roses for CNR threshold values of –17 dB, –22 dB, and –35 dB illustrate that the wind direction at FINO3 predominantly is westerly to south-westerly. It can also be observed that the wind direction gradually become more westerly and south-westerly for increasing CNR threshold values. It is in agreement with [33] that the wind rose for high wind speed generally shifts towards westerly and south-westerly directions. The effect can be observed in all the cases shown in Figure 8—when only the wind observations from the wind lidar at 126 m are filtered with the CNR threshold (upper panels), when it is applied for the wind-vane measurements at 100 m (second upper panels) and when the whole profile is filtered with the CNR threshold value (Figure 8 second lower panels). Excellent agreement is observed between the wind roses for the wind lidar measurements at 126 m and the wind vane measurements at 100 m. By comparing wind roses for the wind lidar measurements at 626 m (lowest panels) and 126 m veering in the wind direction is observed, which underlines that a wind lidar is an excellent instrument for measuring of directional wind shear.

**Figure 9:**
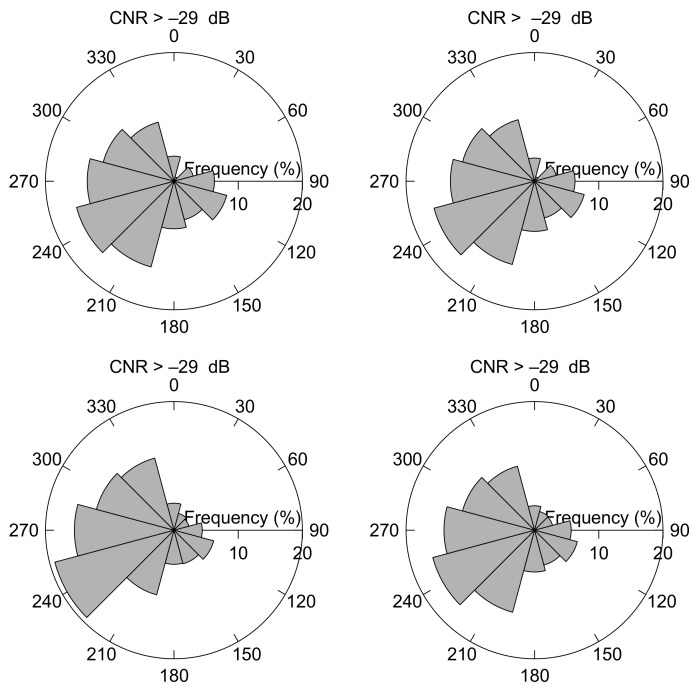
Wind roses for CNR >–29 dB. Upper left panel is the wind rose at 126 m when filtering the wind lidar measurements at 126 m, upper right shows the concurrent wind rose for the vane measurements at 100 m, lower left panel is the wind rose at 626 m for measurements filtered at 626 m, and lower right panel when the CNR threshold is applied to the profile between 126 m and 626 m.

It can be observed in Figure 9 that the wind roses for a CNR threshold of –29 dB turned out to be almost indistinguable from the wind roses for –35 dB. 

## 4. Discussion

A full year of measurements of the wind profile in a windy climate performed at a marine site in the North Sea has been analyzed. The measurements were carried out with a ~ 600 m range wind Doppler lidar in combination with a 100 m tall meteorological mast. The analysis was carried out with observations of wind profiles and CNR values from a WLS-70 Doppler Wind lidar from Leosphere.

**Figure 10:**
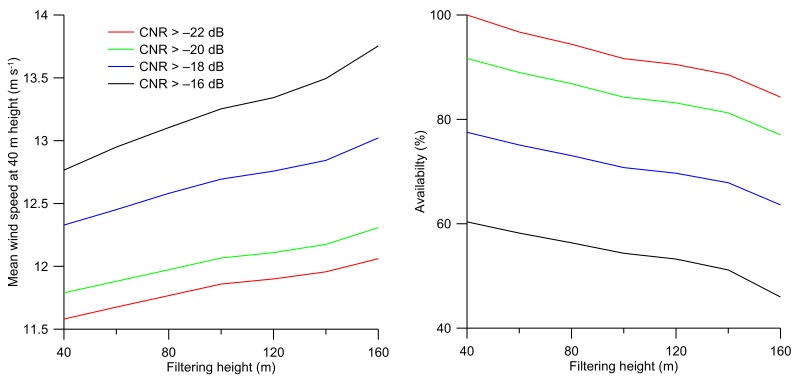
Mean wind speed at 40 m height (left panel) and availability of the observations at 40 m (right panel) plotted as a function of the filtering height. An availability of 100% corresponds to the total number of measurements at 40 m with a CNR >–22 dB.

To assess if the findings are specific for the device used here, it is worth discussing if the effects can be observed in observations from other types of lidars and locations. A similar dependence on the CNR threshold was found applying a S200-Leosphere wind lidar in a study [34] of horizontal (Figure 37 in [35]) and vertical profiles of the wind speed that was performed over the North Sea. As part of this activity, vertical profiling of the wind speed was also carried out with a WLS7 version1 wind lidar (make Leosphere) that was operating at the west coast of Denmark from the beginning of November 2015 to the end of February 2016. The factory CNR threshold setting was –25 dB. Figure 10 shows the mean wind speed at 40 m for a number of CNR threshold values as a function of the filtering height, i.e., the height up to which concurrent measurements fulfill the CNR threshold requirement. It can be observed that the mean wind speed increases when the CNR threshold value is increased, which is in agreement with the findings reported here for the observations at FINO3. The figure also illustrates the availability of the measurements as a function of the CNR threshold value are plotted as a function of the filtering height. We note that similar trends as a function of the CNR threshold and filtering height during the same campaign were observed using a WLS7 version 2 wind lidar (not shown).

A proper mean wind speed estimate should be based on a time-series of wind speed measurements which have data evenly distributed in time, and there should not be extended periods of lacking data. This is especially so if there are variations in the wind conditions, such as missing data from periods with pronounced seasonal variations as are typical for, e. g. the westerlies with high wind speeds during fall, or systematic missing measurements from, for example, night conditions where the wind regime over lands is driven by different physical processes compared to daytime conditions. A typical example is the analysis of the FINO3 data presented here. The resulting mean wind speed is not representative for a yearly average because of the lack of data in July and poor coverage in August and September, therefore, there will be relatively more weight on the measurements in winter and spring, which will cause a bias in the estimation of the real annual mean wind speed. Filtering the observations with a high CNR threshold value favors conditions with high wind speeds resulting in a time-series of unevenly distributed data, and, therefore, will provide a biased estimate of the real mean wind speed for the actual period. It is, therefore, crucial when estimating the mean wind speed over a prescribed period (e.g., annual or seasonal) that the measurements are evenly distributed in time and that measurements are not lacking for extended periods where the wind speed conditions can be climatological different.

**Figure 11:**
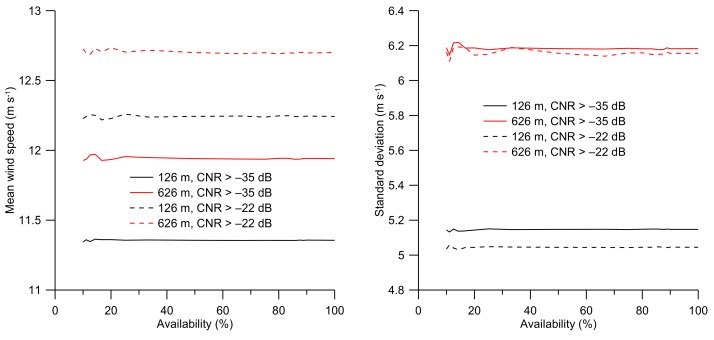
Left panel shows the mean wind speed as a function of data availability for evenly spaced data in time for CNR threshold values of –22 dB and –35 dB, and for 126 m and 626 m height levels. The right panel shows the standard deviation of the observations for the same combinations of observation levels and CNR threshold values.

The error in estimating the mean wind speed over a given period is a function of data availability and data quality. Having more data by lowering the CNR threshold will ensure more samples to better estimate the mean wind speed, but the additional measurements will have a higher uncertainty and may contribute to the error in the estimated mean wind speed and shape of the wind speed distribution. This effect was investigated on the off-shore data from FINO3, and the results are presented in Figure 11 as a function of the availability if the data. It can be observed that the mean wind speed is a weak function of the availability of the measurements. This signifies that a high availability is not essential for the estimate of the mean wind speed as long as the measurements are evenly distributed. Figure 11 right panel illustrates that the standard deviation of the wind speed distributions when applying CNR threshold values of –22 dB and –35 dB respectively are only marginally different (~2%) at 126 m and near identical (~0.4%) at 626 m. This is in agreement with Figure 4 where it can be observed that the distributions when applying CNR threshold values of –22 dB and –35 dB are near identical at both at 126 m and 626 m except that the wind speed distribution is shifted towards higher values when the –22dB CNR threshold is applied as compared to a CNR threshold value of –35 dB. The addition of measurements with a low CNR and, thus, higher uncertainty have only a minor effect on the standard deviation of the wind speed distribution (Figure 7), but the addition of measurements with low CNR have a pronounced effect on the estimated mean wind speed (Figure 5).

Because the number of observations with a prescribed CNR threshold or factory setting decreases with height, a CNR filtering is inevitable (see Figure 10 right panel). Therefore, the effect should be investigated when measuring wind climatologies with wind lidars at several heights. It is presently not known how this affects the estimated wind speed and wind direction profiles due to lack of observations that can be used for comparison from meteorological towers (e.g., cup anemometer and wind vane) at the relevant heights.

It is found in this study that wind lidar measurements in a marine environment like the North Sea preferably should be carried out with a Carrier-to-Noise-Ratio threshold of –29 dB or lower to obtain a near threshold independent estimate of the mean wind speed and wind rose. Here we deal with the marine atmosphere only, but the sensitivity to the CNR threshold value as found by [31] also to apply to rural, coastal, and urban locations. The generally accepted value of –22 dB is too high at these locations, and it is suggested (see Figure 4 in [31]) also to use the –29 dB or lower as a CNR threshold, or alternatively use all valid observations, until further evidence has been obtained for coastal and on-shore conditions.

Although the CNR threshold of –29 dB was found applicable for this lidar, the effect of filtering should always be investigated when deriving wind climatologies from wind lidar observations. Presently, it is hypothesized that the bias is not likely caused by instrument design but, in part, can be ascribed to changes in the aerosol content in the air, which in this study includes particles from sea spray as well as clouds in agreement with Aitkin et al. [28] where strong correlation was found between aerosol backscatter as measured by a ceilometer and the CNR during an experiment in Boulder, U.S. It remains, however, to be investigated if there are any additionally dependence on pulse repetition frequency, pulse averaging, pulse power, pulse width and other parameters as it is the case for the estimate of the uncertainty in the velocity measurements. Manninen et al. [25] and Vakkari et al. [26] presented a novel post-processing algorithm that performs a correction for the instruments background noise. The algorithm which still is in a trial stage has been developed for a Halo Doppler wind lidar and successfully applied to measurements carried out in Finland. It is not clear how the CNR threshold filtering will affect the wind speed climatology when a post-processing algorithm is applied, but it likely will shift the bias towards lower CNR threshold values.

## 5. Conclusions

Based on one year of observations performed at the FINO3 research platform in the North Sea, it was found that the wind speed distribution is shifted towards higher values at all height levels (126 m to 626 m) when the Carrier-to Noise-Ratio threshold value is changed from –35 dB to –22 dB. As a consequence, the wind power density is shifted even more (32%) applying the CNR threshold for the whole profile (126 m to 626 m) and somewhat less (18%) when imposing the CNR threshold at 126 m only. Furthermore, the wind rose is shifted towards westerly and south-westerly directions for increasing CNR threshold values. 

Consequently, the Carrier-to Noise-Ratio threshold filtering should be done cautiously when creating a wind climatology. Here it is suggested that the lower limit for the Carrier-to-Noise-Ratio of the observations should be –29 dB or less, to obtain a threshold independent estimate of the mean wind speed and wind rose. Alternatively, all valid observations should be used for the analysis. Although this study is specific for the synoptic conditions in the North Sea, we suggest that for a representative estimation of the wind resource with wind lidars, the effect of the CNR threshold filtering on the wind distribution should be studied when the lidar recovery rate is less than 100%. 

## References

[1] Global Wind Energy Council (2016). Global Wind Energy Outlook 2016.

[2] Global Wind Energy Council (2018). Global Wind Report, Annual Market Update 2017.

[3] Troen I., Petersen E. (1988). European Wind Atlas.

[4] Carvalho D., Rocha A., Gómez-Gesteira M., Silva Santos C. (2014). Offshore wind energy resource simulation forced by different reanalyses: Comparison with observed data in the Iberian Peninsula. Appl. Energy.

[5] Durante F., Westerhellweg A. (2012). A comparison of a mesoscale model with FINO measurements in the German Bight and the Baltic Sea. DEWI Mag..

[6] Floors R., Vincent C.-L., Gryning S.E., Peña A., Batchvarova E. (2013). The wind profile in the coastal boundary layer: Wind lidar measurements and numerical modelling. Bound.-Layer Meteorol..

[7] Gryning S.E., Batchvarova E., Brümmer B., Larsen S.E. (2007). On the extension of the wind profile over homogeneous terrain beyond the surface layer. Bound.-Layer Meteorol..

[8] Steyn D.G., McKendry I.G. (1988). Quantitative and qualitative evaluation of a three-dimensional mesoscale numerical model simulation of a sea breeze in complex terrain. Mon. Weather Rev..

[9] Suomi I., Gryning S.E., O’Connor E.J., Vihma T. (2017). Methodology for obtaining wind gusts using Doppler lidar. Q. J. R. Meteorol. Soc..

[10] Peña A., Floors R., Sathe A., Gryning S.E., Wagner R., Courtney M.S., Lársen X.G., Hahmann A.N., Hasager C.B. (2016). Ten years of boundary-layer and wind-power meteorology at Høvsøre, Denmark. Bound.-Layer Meteorol..

[11] Brümmer B., Lange I., Konow H. (2012). Atmospheric boundary layer measurements at the 280 m high Hamburg weather mast 1995–2011: Mean annual and diurnal cycles. Meteorol. Z..

[12] Brümmer, B., Schultze. M (2015). Analysis of a 7-year low-level temperature inversion data set measured at the 280 m high Hamburg weather mast. Meteorol. Z..

[13] Borsche M., Kaiser-Weiss A.K., Kaspar F. (2016). Wind speed variability between 10 and 116 m height from the regional reanalysis COSMO-REA6 compared to wind mast measurements over Northern Germany and the Netherlands. Adv. Sci. Res..

[14] Van Ulden A.P., Wieringa J. (1996). Atmospheric boundary layer research at Cabauw. Bound.-Layer Meteorol..

[15] Barthelmie R.J., Pryor S.C. (2003). Can satellite sampling of offshore wind speeds realistically represent wind speed distributions?. J. Appl. Meteorol..

[16] Chang R., Zhu R., Badger M., Hasager C.B., Zhou R., Ye D., Zhang X. (2014). Applicability of Synthetic Aperture Radar Wind Retrievals on Offshore Wind Resources Assessment in Hangzhou Bay, China. Energies.

[17] Nygaard N.G., Newcombe A.C. (2018). Wake behind an offshore wind farm observed with dual Doppler radars. Proceedings of the Science of Making Torque from Wind (TORQUE 2018), Milan, Italy, 20–22 June 2018.

[18] Cariou J.P. (2013). Pulsed Lidars. Remote Sensing for Wind Energy.

[19] Gryning S.E., Mikkelsen T., Baehr C., Dabas A., Gómez P., O’Connor E., Rottner L., Sjöholm M., Suomi I., Vasiljević N., Kariniotakis G. (2017). Measurement methodologies for wind energy based on ground-level remote sensing. Renewable Energy Forecasting, from Models to Applications.

[20] Frehlich R. (1996). Simulation of coherent Doppler lidar performance in the weak-signal regime. J. Atmos. Ocean. Technol..

[21] O’Connor E.J., Illingworth A.J., Brooks I.M., Westbrook C.D., Hogan R.J., Davies F., Brooks B.J. (2010). A method for estimating the turbulent kinetic energy dissipation rate from a vertically-pointing Doppler lidar, and independent evaluation from balloon-borne in-situ measurements. J. Atmos. Ocean. Technol..

[22] Gryning S.E., Batchvarova E., Floors R., Peña A., Brümmer B., Hahmann A.N., Mikkelsen T. (2014). Long-term profiles of wind and Weibull distribution parameters up to 600 m in a rural coastal and an inland suburban area. Bound.-Layer Meteorol..

[23] Suomi I., Gryning S.E., Floors R., Vihma T., Fortelius C. (2015). On the vertical structure of wind gusts. Q. J. R. Meteorol. Soc..

[24] Frehlich R. (2001). Estimation of velocity error for Doppler lidar measurements. J. Atmos. Ocean. Technol..

[25] Manninen A.J., O’Connor E.J., Vakkari V., Petäjä T. (2016). A generalised background correction algorithm for a Halo Doppler lidar and is application to data from Finland. Atmos. Meas. Tech..

[26] Vakkari V., Manninen A.J., O’Connor E.J., Schween J.H., Van Zyl P.G. (2018). A novel post-processing algorithm for Halo Doppler lidars. Atmos. Meas. Tech..

[27] Beck H., Kühn M. (2017). Dynamic Data Filtering of Long-Range Doppler LiDAR Wind Speed Measurements. Remote Sens..

[28] Aitkin M.L., Rhodes M.E., Lundquist J.E. (2012). Performance of a wind-profiling lidar in the region of wind turbine rotor disks. J. Atmos. Ocean. Technol..

[29] Fujii Y., Yamashita J., Shikata S., Saito S. (1998). Incoherent optical heterodyne detection and its application to air pollution detection. Appl. Opt..

[30] Fujii T., Fukuchi T. (2005). Laser Remote Sensing.

[31] Gryning S.E., Floors R., Pena A., Batchvarova E., Brümmer B. (2016). Weibull Wind-Speed Distribution Parameters Derived from a Combination of Wind-Lidar and Tall-Mast Measurements Over Land, Coastal and Marine Sites. Bound.-Layer Meteorol..

[32] Peña A., Gryning S.E., Floors R. (2015). Lidar observations of marine boundary-layer winds and heights: A preliminary study. Meteorol. Z..

[33] Cappelen J., Jørgensen B. (1999). Observed Wind Speed and Direction in Denmark—With Climatological Standard normal, 1961–90.

[34] Floors R., Peña A., Lea G., Vasiljević N., Simon E., Courtney M. (2016). The RUNE Experiment—A Database of Remote-Sensing Observations of Near-Shore Winds. Remote Sens..

[35] Floors R., Hahmann A., Peña A., Karagali I. (2016). Estimating Near-Shore Wind Resources. DTU Wind Energy Report E-0116.

